# Add-on efgartigimod in myasthenic crisis: a promising treatment option

**DOI:** 10.3389/fneur.2025.1696976

**Published:** 2026-01-08

**Authors:** Fangyi Shi, Jing Wang, Jiaxin Chen, Rong Lai, Li Feng, Hongyan Zhou, Xunsha Sun, Cunzhou Shen, Jiezhen Feng, Xin Huang, Huiyu Feng, Haiyan Wang

**Affiliations:** 1 Department of Neurology, The First Affiliated Hospital, Sun Yat-sen University, Guangzhou, China; 2 Guangdong Provincial Key Laboratory of Diagnosis and Treatment of Major Neurological Diseases, Guangzhou, China; 3 Department of Neurosurgical Intensive Care Unit, The First Affiliated Hospital of Sun Yat-sen University, Sun Yat-sen University, Guangzhou, China

**Keywords:** myasthenia gravis, efgartigimod, myasthenic crisis, intensive care unit, Mechanical ventilation, fast-acting treatment

## Abstract

**Background:**

In myasthenia crisis (MC), plasma exchange (PE) and intravenous immunoglobulin (IVIG) are confirmed effective treatment options, but PE may not be available in time, and the response rate to IVIG is not always satisfactory. This study aimed to investigate whether addition of efgartigimod confers benefits in patients with MC.

**Methods:**

This real-world retrospective pilot study examined MC patients admitted to the First Affiliated Hospital of Sun Yat-sen University between August 2023 and May 2024, who were categorized into two groups: the traditional immunotherapy group (*n* = 14) and the add-on efgartigimod group (*n* = 9). In the efgartigimod group, patients received 20 mg/kg efgartigimod (*n* = 9), administered on the first and fifth days.

**Results:**

Totally 23 patients were recruited in the study. Total hospital stays, MV duration, and non-invasive ventilation time were shorter in the efgartigimod group compared with the traditional immunotherapy group; however, these differences were not statistically significant (*p* > 0.05). We subsequently applied a generalized estimating equation (GEE) to the patients over an 8-week period and observed a reduction in Myasthenia Gravis Activities of Daily Living (MG-ADL) scores in both groups; however, no significant differences were found within the groups (*p* > 0.05). Survival analysis was also conducted, revealing that the addition of efgrtigimod allowed patients to reach the MSE state more quickly, which is statistically significant (*p* = 0.0499). Additionally, three patients with MC who only received high-dose efgartigimod treatment all achieved favorable treatment outcomes.

**Conclusion:**

This suggests that efgartigimod may serve as an alternative option for MC patients or as a rescue treatment option for refractory individuals.

## 1 Introduction

Myasthenia crisis (MC), a rapidly progressing condition affecting respiratory and bulbar muscles, is the leading cause of death in individuals with myasthenia gravis (MG) (1, 2). MC imposes a substantial burden on individuals and society, severely decreasing the quality of life of patients. MC occurs in 10–20% of MG patients, the majority of whom develop MC within 2 years of MG diagnosis (3). Currently, MC treatment primarily involves intravenous high-dose methylprednisolone (IVMP), plasmapheresis (PE), and intravenous immunoglobulin (IVIG) (1, 2, 4), commonly referred to as fast-acting treatment. Fast-acting treatment is defined as the aggressive application of non-oral immunotherapies, including PE, IVIG, and/or IVMP as first-line treatments, as recommended by various guidelines for MG (5).

Efgartigimod is an Fc receptor inhibitor that facilitates the clearance of pathogenic antibodies by binding to Fc receptors, thereby alleviating clinical symptoms (6). The efficacy and safety of efgartigimod in patients with generalized myasthenia gravis (GMG) have been demonstrated in phase I, II, and III clinical trials (6–8), which have consistently excluded patients experiencing MC. At present, only a limited number of case reports have demonstrated the efficacy and safety of add-on efgartigimod in patients with refractory myasthenic crisis (RMC) showing poor response to traditional fast-acting treatment (9–15). After switching to efgartigimod, these patients were successfully weaned off mechanical ventilation (MV), with no significant adverse reactions, indicating good efficacy and safety for efgartigimod.

Those case reports solely focused on individual use or addition of efgartigimod, thereby failing to ascertain whether efgartigimod confers any advantages to MC patients compared to traditional immunotherapy treatment. This retrospective real-world pilot study aimed to shed some light on it.

## 2 Materials and methods

### 2.1 Patients

Inclusion criteria were: (1) >18-years-old; (2) diagnosis of MG made by satisfying the following three criteria: (a) fluctuating muscle weakness; (b) positive neostigmine test, decreased compound motor action potential shown in slow repetitive nerve stimulation, or positive serum anti-acetylcholine receptor (AChR) antibody; (c) other causes of skeletal muscle weakness excluded; (3) fast-acting treatment (including IVMP, IVIG and PE) administered in the Department of Neurology, the First Affiliated Hospital of Sun Yat-sen University from August 2023 to May 2024; (4) All patients in the efgartigimod group received at least one cycle of efgartigimod treatment; (5) All participants in the study met the diagnostic criteria for myasthenic crisis. The international definition of manifest MC is worsening of myasthenic weakness requiring intubation or noninvasive ventilation to avoid intubation, except when these measures are applied in routine postoperative management (4).

### 2.2 Study design

This was a retrospective real-world pilot study including all patients diagnosed with MG who were admitted to the First Affiliated Hospital of Sun Yat-sen University between August 2023 and May 2024. Patients were divided into two groups: the add-on efgartigimod group (*n* = 9) and the traditional immunotherapy group (*n* = 14), based on whether efgartigimod was added. The traditional immunotherapy group received fast-acting treatment with IVMP (1 g for 5 days), IVIG (0.4 g/kg for 5 days), or PE (1.0 plasma volume exchanges with 5% albumin replacement fluid, administered every other day for a total of five sessions). In the add-on efgartigimod group (*n* = 9), patients were administered 20 mg/kg efgartigimod on days 1 and 5.

The primary outcomes of this investigation included the length of stay in the intensive care unit (ICU) and MV duration. Additionally, this study examined the total length of hospital stay, non-invasive ventilation duration, intubation duration, and changes in Myasthenia Gravis Activities of Daily Living (MG-ADL) score from baseline (at the time of ventilation initiation) to the time of extubation and at various times within 8 weeks post-extubation.

Prior to efgartigimod administration the patients may have been administered other treatments such as IVMP, PE, and IVIG. The doses of oral medications, including tacrolimus and azathioprine, were gradually reduced or kept constant throughout the treatment period according to the primary disease course.

MG-ADL scores were prospectively collected at baseline (when on a ventilator) and weekly after extubation until 8 weeks. In instances where patients were unable to provide MG-ADL scores at baseline—due to conditions such as intubation, sedation, or altered consciousness—retrospective data were collected from specialists, caregivers, or family members who were most familiar with the patient’s condition. This data, combined with medical records and clinical observations from healthcare professionals, would yield the corresponding scores.

Patient data were obtained from medical records specific to MG, antibody status, history of thymoma and thymectomy, and MG-specific treatment at the start of fast-acting treatment. Meanwhile, the time from admission to successful weaning and total hospital stay were retrieved. Minimal symptom expression (MSE) was defined as an MG-ADL score of 0 or 1 (16, 17).

### 2.3 Statistical analysis

Data were categorized into quantitative and qualitative variables, which were summarized and analyzed separately (i.e., mean ± standard deviation, median and range, count and percentage). Depending on distribution, the t-test or the Mann–Whitney U rank-sum test was employed to analyze continuous data, assessing the differences between the two patient groups regarding disease duration, age, MG-ADL score, length of ICU stays, and MV duration. Normally distributed continuous variables are presented as mean ± standard deviation, while non-normally distributed data are expressed as median (interquartile range). Effect sizes were calculated using the coefficient of rank correlation. According to Cohen’s (1988) standards, *r* = 0.10 indicates a small effect, 0.30 a medium effect, and 0.50 a large effect. The chi-square test or the Fisher’s exact probability method was utilized to determine differences in categorical variables, including gender, thymic pathology, safety, and other relevant factors. Changes in MG-ADL scores from baseline (when on a ventilator) to 8 weeks post-extubation were calculated using a generalized linear regression model. A *p*-value of less than 0.05 was considered statistically significant. Furthermore, a *post hoc* power analysis was performed for inter-group comparisons to better interpret nonsignificant results. All statistical analyses were conducted with the SPSS software (version 26, IBM Corporation).

## 3 Results

### 3.1 Clinicodemographic characteristics of patients with MC

**Figure 1:**
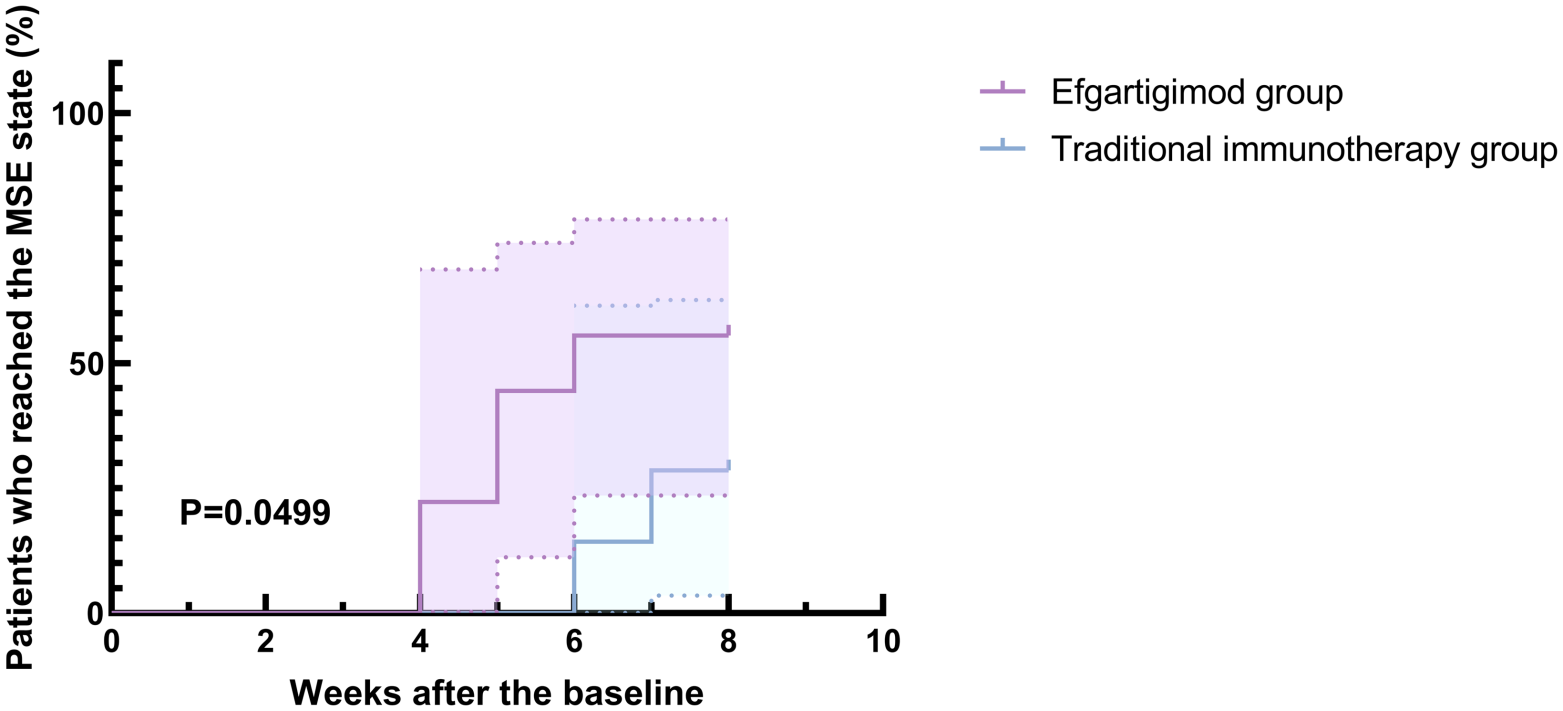
Percentages of patients reaching the MSE status in the efgartigimod and traditional immunotherapy groups. Kaplan–Meier survival curves for add-on efgartigimod and traditional immunotherapy in patients with myasthenia gravis. Significant difference was observed between the treatment groups (*p* = 0.0499).

Totally 23 eligible individuals were included, of whom 9 received efgartigimod based on other fast-acting treatment regimens (*n* = 9) and 14 only received traditional immunotherapy treatment (*n* = 14) (Figure 1). Demographic data are presented in Table 1, with all groups remaining balanced for all aspects. The study population was predominantly female (52.2%), with ages of 47.17 ± 15.72 years and disease durations of 21 (4, 42) months. Most patients (78.26%) tested positive for acetylcholine receptor (AchR) antibodies, while five (21.74%) were positive for muscle-specific kinase (MuSK) antibodies. Within the efgartigimod group, 5 patients (45.5%) had concurrent thymoma, with 4 administered thymectomy. Conversely, in the traditional immunotherapy treatment group, 9 patients (64.3%) had concurrent thymoma, of whom 6 underwent thymectomy. The baseline immunotherapy regimens were well matched overall between the two groups. Of all patients, 21.7% received only corticosteroids, 43.5% were treated with prednisone in combination with non-steroidal immunosuppressive therapy (NSIST), and 13% received no corticosteroids or immunosuppressants (patients originally presenting with MC) (Table 1).

**Table 1:** Clinicodemographic characteristics of patients with MC.

	All patients			
	Total	Efgartigimod group	Traditional immunotherapy group	*p* value
	*n* = 23	*n* = 9	*n* = 14	
Age, years	47.17 ± 15.72	52.44 ± 14.82	43.79 ± 15.87	0.204
Sex				1.0
Female	12 (52.2%)	5 (55.6%)	7 (50%)	
Male	11 (47.8%)	4 (44.4%)	7 (50%)	
MG duration, months	21 (4, 42)^#^	36 (12, 168)	12.5 (3, 39)	0.164
Antibodys				1.0
Acetylcholine receptor antibody-positive	18 (80%)	7 (77.8%)	12 (85.7%)	
MusK-ab-positive	5 (20%)	2 (22.2%)	2 (14.3%)	
Antibody negative	0	0	0	
Thymoma	14 (60.9%)	5 (55.6%)	9 (64.3%)	0.623
Thymectomy	10 (43.5%)	4 (44.4%)	6 (42.9%)	1.0
Myasthenia gravis therapy at baseline				
Only steroid	5 (21.7%)	2 (22.2%)	3 (21.4%)	1.0
Steroid and NSIST	10 (43.5%)	3 (33.3%)	7 (50%)	0.669
No steroid or NSIST	3 (13%)	1 (11.1%)	2 (14.3%)	1.0
Fast-acting treatment				
IVMP	14 (60.9%)	6 (66.7%)	8 (57.1%)	1.0
PE	10 (43.5%)	1 (11.1%)	9 (61.3%)	0.029
IVIG	16 (69.6%)	6 (66.7%)	10 (71.4%)	1.0
Neither	3 (13%)	3 (33.3%)	0	

MG, myasthenia gravis; MuSK-Ab, muscle-specific tyrosine kinase antibodies; IVIG, intravenous immunoglobulin; PE, plasma exchange; IVMP, intravenous high-dose methylprednisolone; NSIST, non-steroidal immunosuppressive therapy.

#: Normally distributed continuous variables are presented as mean ± standard deviation, while non-normally distributed data are expressed as median (interquartile range).

Regarding fast-acting treatment, most patients opted for IVMP or IVIG, with a significantly higher number of patients receiving PE in the traditional immunotherapy group compared with the efgartigimod group. In the traditional immunotherapy group, the frequency of fast-acting treatment regimens (IVMP, IVIG, PE) utilized by the patients was generally consistent. Of these patients, five received a combination of two fast-acting treatment regimens, three received a combination of three regimens, and six had a single treatment regimen.

In the efgartigimod group (*n* = 9), three patients received only efgartigimod; of the remaining 6 patients, the majority (*n* = 4) initiated efgartigimod following the ineffectiveness of other rapid-acting therapeutic options in facilitating ventilator weaning. One patient received IVMP concurrently with efgartigimod infusion. One patient in the efgartigimod group started efgartigimod treatment immediately following the diagnosis of MC (this patient originally presenting with MC) whose response to efgartigimod was inadequate, requiring sequential treatment with IVIG and IVMP.

### 3.2 Clinical effectiveness of efgartigimod for the treatment of MC

As shown in Table 2, length of MV duration, hospital stay, noninvasive positive pressure ventilation (NIPPV) time, and were all shorter in the efgartigimod group compared with the traditional immunotherapy group, although the differences were not statistically significant.

**Table 2:** The length of ICU stays, Mechanical Ventilation duration, hospital stays, NIPPV time, and intubation duration in high-dose efgartigimod group and traditional immunotherapy group.

	Efgartigimod group	Traditional immunotherapy group	*p* value	*r* ^a^
Hospital stay, day	22 (18, 24)	24 (16, 47)	0.486	−0.15
ICU stay, day	19 (18, 24)	15.5 (11, 35)	0.526	−0.14
Mechanical Ventilation duration, day	18 (16,23)	19 (10, 37)	0.89	−0.03
Intubation time, days	17 (15, 21)	14.5 (6, 37)	0.988	−0.01
NIPPV time, day	3.55 ± 3.68 (0.73, 6.38)	5.14 ± 4.82 (2.36,7.92)	0.524	−0.14

ICU, intensive care unit, NIPPV, noninvasive positive pressure ventilation.

^a^Effect sizes were calculated using the coefficient of rank correlation. According to Cohen’s (1988) standards, *r* = 0.10 indicates a small effect, 0.30 a medium effect, and 0.50 a large effect. All indicators showed small effects.

At the 8-week follow-up, 88.9% of patients (8/9) in the efgartigimod group and 85.7% (12/14) in the traditional immunotherapy group were successfully weaned off the ventilator, with no statistical significance. While, the last two patients—one in the traditional immunotherapy group and one in the efgartigimod group—still required intermittent small non-invasive ventilators to support their daily activities. Additionally, one patient in the traditional immunotherapy group remains hospitalized. During the follow-up period, 4 patients in the traditional immunotherapy group and 5 in the efgartigimod group achieved the MSE status, and Kaplan–Meier survival curve comparison between the two groups indicated significant difference (*p* = 0.0499) (Figure 1).

**Figure 2:**
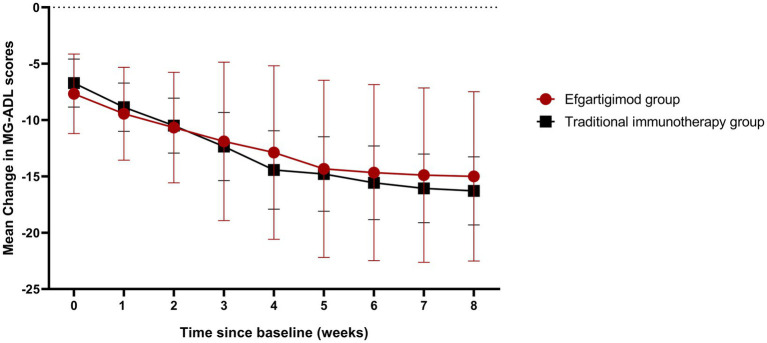
Changes in MG-ADL scores from baseline (when on a ventilator) to 8 weeks post-extubation in both groups.

The rank-sum test revealed no differences in MG-ADL score changes before and after ventilation between the efgartigimod and traditional immunotherapy group [efgartigimod group 18.22 ± 4.21 (14.99, 21.46) vs. traditional immunotherapy group 19.71 ± 2.52 (18.26, 21.17), *p* = 0.298]. The generalized estimating equation (GEE) indicated that the changes from baseline (when patients were on a ventilator) to 8 weeks post-discharge demonstrated a continued decline in MG-ADL scores in both groups over time (efgartigimod group Wald *χ*^2^ time = 2.026E+13, *p* < 0.05; traditional immunotherapy group Wald *χ*^2^ time = 1082.973, *p* < 0.05). The changes in MG-ADL scores from baseline did not differ significantly between the two treatment groups throughout the study. A graphical representation of these changes is provided below (Figure 2).

**Figure 3:**
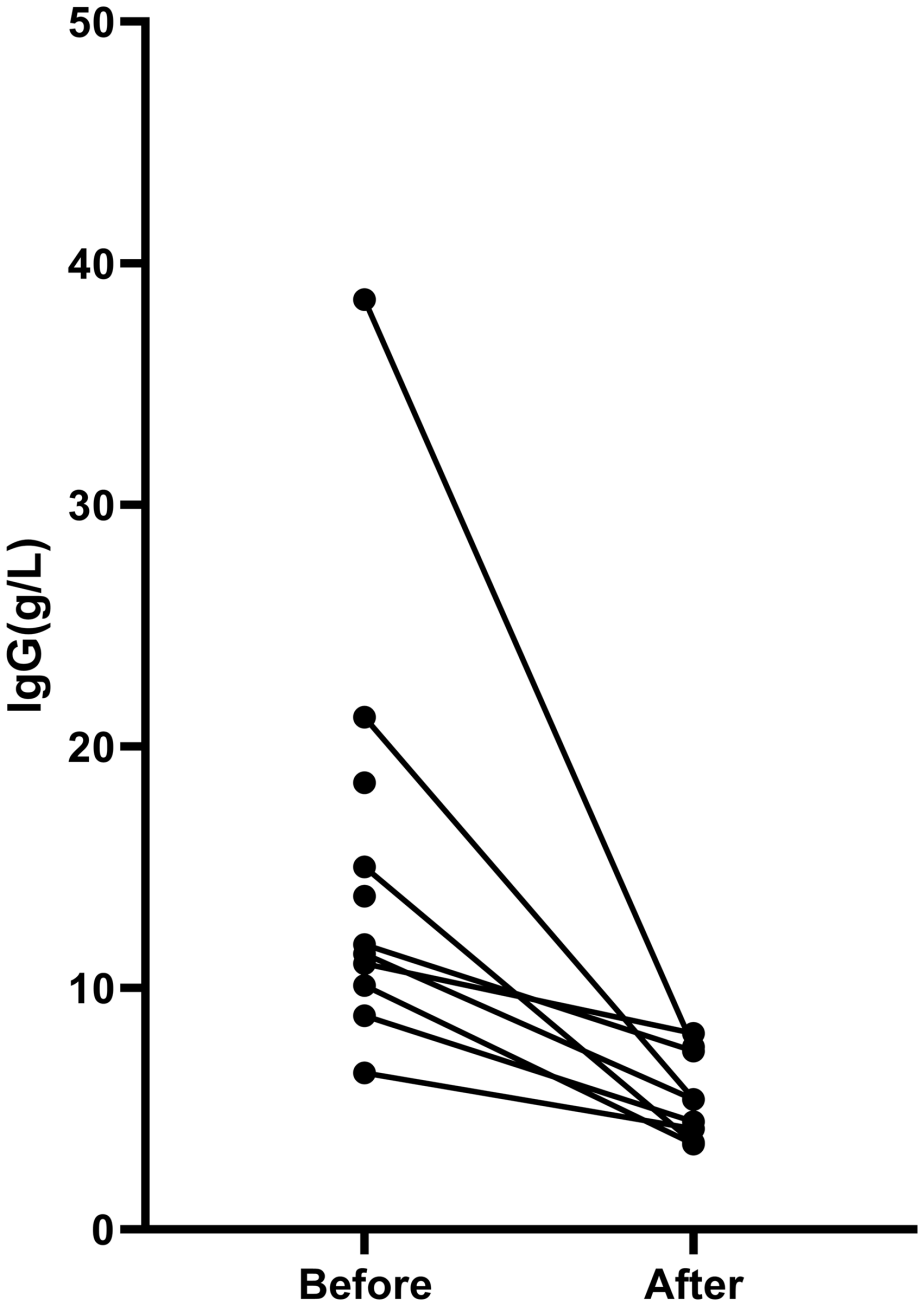
Changes in immunoglobulin G (IgG) levels after one cycle of efgartigimod treatment in the efgartigimod group versus pretreatment values.

Due to insufficient data in the traditional immunotherapy group, we only analyzed the changes in IgG levels within the efgartigimod group following drug treatment. These levels decreased from 14.79 ± 9.78 mmol/L prior to treatment to 5.74 ± 1.73 mmol/L thereafter (Figure 3).

We conducted a *post hoc* power analysis using a simulation-based Pearson correlation test for the primary outcome measures, such as ICU length of stay and duration of mechanical ventilation. The results showed that the statistical power was 9.42 and 5.04%, respectively.

### 3.3 Safety of efgartigimod in the treatment of MC

Adverse events in the efgartigimod group included allergic reactions (1 case), gastrointestinal reactions (6 cases), headaches (1 cases), hypoalbuminemia (4 cases), fever (3 cases), thrombocytopenia (1 case), and infections (3 cases, comprising 1 viral, 1 fungal, and 1 bacterial infections). Notably, one patient in the efgartigimod group had low platelet count during PE, requiring urgent cessation of the procedure. Additionally, one patient developed a rash, attributed to atopic dermatitis/itching, which improved following moisturizing and allergy treatment.

In the traditional immunotherapy treatment group, adverse events included gastrointestinal reactions (7 cases), hypoalbuminemia (7 cases), fever (5 cases), and infections (5 cases, including 1 viral, 2 fungal, and 2 bacterial infections). One patient developed a rash, suspected to be associated with a viral infection, while another had upper gastrointestinal bleeding, potentially linked to IVMP.

## 4 Discussion

The primary goal of MC treatment is to rapidly eliminate antibodies in order to quickly alleviate symptoms. IVMP, IVIG, and PE are all traditional, fast-acting treatment options that are widely utilized in patients with MC. However, IVMP, which is currently the most commonly applied regimen in China, poses a risk of transient symptom exacerbation and is not recommended without ensuring adequate ventilatory support. Moreover, the substantial side effects of high-dose steroids, e.g., severe infections, hyperglycemia, gastrointestinal reactions, skin changes, and obesity, should be considered. Besides patients with MC testing positive for muscle-specific kinase (MUSK) antibodies, randomized controlled studies have confirmed that the efficacies of IVIG and PE, commonly utilized as traditional, fast-acting treatment options in patients with MC, are comparable (18). However, PE does not solely remove IgG antibodies, but also results in reduced serum immunoglobulin levels and increased risk of moderate to severe AEs. Furthermore, PE is considered an invasive procedure that must be performed in hospitals with trained, specialized nursing staff and adequate equipment, greatly limiting its availability in primary hospitals. Compared with PE, IVIG is more convenient and is often preferred; however, its practicality is constrained by high cost and a limited supply of voluntary blood donors, while some patients also have a poor response to IVIG.

Efgartigimod, a novel Fc receptor inhibitor, has received approval for the treatment of GMG in several countries and is recommended for severe GMG and RMG (15), including the United States, China, and Japan (22). Post-hoc analysis of the ADAPT trial suggested that efgartigimod significantly enhances the functions of bulbar and respiratory muscles compared with the control group, with notable improvements in MG-ADL and quantitative myasthenia gravis (QMG) scores (19). Additionally, the effects of efgartigimod begin within the first 1–2 weeks following the initial infusion, demonstrating significant rapidity and efficacy in rescuing MC cases. The guidelines for impending MC in China have already incorporated these recommendations (20). Currently, multiple case reports have confirmed the efficacy and safety of efgartigimod in patients with MC (10–15). In these reports, most patients exhibited poor responses to IVIG or PE, which is termed RMC, prompting efgartigimod addition. Then, they successfully recovered from MC following treatment with efgartigimod. Additionally, some case reports indicated a gradual level decrease in CD19+ B cells and CD4+ T cells while using efgartigimod (9, 11). This evidence collectively supports the use of efgartigimod in patients with MC. This study aimed to investigate whether addition of efgartigimod could confer benefits in MC patients versus traditional immunotherapy.

Notably, patients in the efgartigimod group (*n* = 9) received high-dose efgartigimod. The decision to administer a higher dosage with an alternative administration schedule (20 mg/kg efgartigimod on days one and five) derived from the treatment protocol established in an ongoing phase II clinical trial of Guillain-Barré syndrome (GBS) (NCT05701189). Findings from the pharmacodynamic study conducted during the phase I clinical trial of efgartigimod demonstrated that doses ranging from 10 to 25 mg/kg, administered every 4–7 days, do not result in accumulation of toxic effects and are deemed safe (8). Consequently, we considered efgartigimod administration at a dose of 20 mg/kg to be relatively safe in the treatment of MC or pre-crisis state. Furthermore, we posit that the management approach for MC cases differs from that applied for other MG cases, necessitating short-term, fast effective treatment regimens akin to IVIG or PE. Using standard treatment regimens (10 mg/kg once weekly for a total of 4 doses) for MC patients may not be suitable. This new dosing regimen compresses the original 4-week treatment into 5 days, and the single dose was doubled while maintaining the total dosage unchanged, which is expected to decrease treatment duration and facilitate the recovery of patients during exacerbation. Prior research conducted by our institution has demonstrated that efgartigimod is both a safe and effective treatment for patients experiencing MC, assisting them in managing the crisis and achieving the MSE state (21). Although no statistically significant differences were identified versus the traditional immunotherapy group, the high-dose efgartigimod group exhibited shorter ICU stay, Mechanical Ventilation duration, hospital stay, NIPPV time, and intubation duration compared with the traditional immunotherapy treatment group. Furthermore, survival analysis indicates that the adding on efgartigimod can facilitate quicker symptom relief and earlier attainment of the MSE state, with statistically significant results. Therefore, administering high doses of efgartigimod to patients in MC is advantageous.

Meanwhile, three patients in the efgartigimod group received no other fast-acting treatment regimens and were solely administered high-dose efgartigimod, including one patient who tested positive for Musk antibodies. Their total hospital stays were 18 days meanly (10, 18, and 22 days, respectively), with ICU stays of 18 days meanly (2, 18, and 22 days, respectively). Mean MV durations were 15 days (10, 15, and 19 days, respectively), while intubation times were 15 and 21 days, respectively, with one patient not requiring intubation, and mean NIPPV times were 3 days (1, 3, and 10 days, respectively). Patients were weaned off the ventilator after 9 days meanly (2, 9, and 10 days, respectively) of add-on efgartigimod treatment, and ADL scores decreased to 1 or 2 points at the final week of follow-up. Following efgartigimod treatment, the patients were successfully weaned off the ventilator, achieving the endpoint 9 days after efgartigimod treatment, without experiencing moderate to severe adverse effects. This finding indicates that high-dose efgartigimod may serve as a viable option for MC. Overall, further clinical research is required to validate this finding.

In RMC patients, achieving symptom relief with a single fast-acting treatment regimen is often challenging, necessitating the use of multiple therapeutic approaches. It is important to note that PE removes molecules such as albumin, globulin and other medium-to-large molecules, which encompass immunoglobulin and efgartigimod; therefore, concurrent use of these two treatments is not advised within a two-week period following PE. Moreover, IVIG can saturate neonatal Fc receptors, thereby accelerating the clearance of efgartigimod, while efgartigimod similarly expedites the removal of IgG molecules. Given that IVIG is predominantly composed of IgG1 molecules, this interaction results in IVIG clearance as well. Consequently, the reciprocal effects of these fast-acting treatment options require a judicious selection of the treatment plan for MC patients, emphasizing the importance of a rational treatment sequence. For instance, caution is warranted when considering efgartigimod or IVIG administration. Should these interventions prove ineffective, subsequent treatments may undermine prior efforts, leading to increased financial burden and psychological distress for the patient.

In this study, add-on efgartigimod treatment demonstrated no significant therapeutic advantage compared to traditional immunotherapy as for the primary outcomes, likely for the following reasons: 1. Most patients in the efgartigimod group (*n* = 8) received this treatment after failure of other therapies, indicating that these individuals had more severe and complex conditions, which consequently extended treatment duration. 2. The efgartigimod group employed additional treatment compared to the traditional immunotherapy group, which required a certain amount of time, thereby prolonging the overall treatment duration and impacting statistical comparisons of hospital stay and MV duration.

In this investigation, two patients, including one each in the traditional immunotherapy group and efgartigimod group required intermittent non-invasive ventilation therapy 8 weeks post-extubation. However, blood gas analysis in these patients demonstrated no evidence of CO_2_ retention. The challenges faced by these patients in the process of weaning from mechanical ventilation may be attributed to prolonged mechanical ventilation following successful extubation, resulting in ventilator dependence. Furthermore, this situation may also be associated with the requirement for home oxygen therapy. Thus, since these patients have confirmed respiratory muscle involvement, intermittent ventilator use does not necessarily mean that the patient is fully out of MC.

The limitations of this article were as follows; 1. As a retrospective real-world single-center pilot study, sample size calculation was not performed; 2. The analyzed patients lacked data about the changes in antibody titers, and most of them only underwent one cycle of efgartigimod treatment; 3. This study exclusively collected MG-ADL scores in patients with MC in the ICU owing to their sedated condition, potentially resulting in higher (QMG scores). Presently, no tailored scoring system is available for MC patients; 4. It is important to note that this study is based on a small sample size, which may limit the generalizability of the findings. Moreover, the small sample size resulted in low post-hoc power, which may have limited our ability to detect small inter-group differences. A larger sample size or collaboration among multiple teams is needed to improve power; 5. As a real-world point study where treatment allocation was based on physician assessment and patient preference rather than randomization, our study is susceptible to selection bias. The primary aim of this research is to provide new insights into the treatment of MC patients, and further validation through multi-center randomized controlled trials (RCTs) is necessary in the future.

## 5 Conclusion

This retrospective real-world pilot study provides evidence for the efficacy of efgartigimod as an add-on fast-acting treatment option for patients with MC. Although the parameters assessed in this study, including length of ICU stay, Mechanical Ventilation duration, hospital stay, NIPPV time, and intubation time, did not reach statistical significance, add-on efgartigimod group exactly show lower durations. Therefore, efgartigimod could serve as an alternative option for MC patients or as a rescue treatment for refractory cases. Further clinical studies are required to substantiate these findings.

## Data Availability

The raw data supporting the conclusions of this article will be made available by the authors without undue reservation.
